# Site-Specific Integration of Exogenous Genes Using Genome Editing Technologies in Zebrafish

**DOI:** 10.3390/ijms17050727

**Published:** 2016-05-13

**Authors:** Atsuo Kawahara, Yu Hisano, Satoshi Ota, Kiyohito Taimatsu

**Affiliations:** 1 Laboratory for Developmental Biology, Center for Medical Education and Sciences, Graduate School of Medical Science, University of Yamanashi, 1110 Shimokato, Chuo, Yamanashi 409-3898, Japan; satoshio-as@yamanashi.ac.jp (S.O.); ktaimatsu@yamanashi.ac.jp (K.T.); 2 Laboratory for Developmental Gene Regulation, Brain Science Institute, RIKEN, 2-1 Hirosawa, Wako, Saitama 351-0198, Japan; y.hisano@riken.jp

**Keywords:** genome editing, CRISPR/Cas9 system, MMEJ, knock-in, zebrafish

## Abstract

The zebrafish (*Danio rerio*) is an ideal vertebrate model to investigate the developmental molecular mechanism of organogenesis and regeneration. Recent innovation in genome editing technologies, such as zinc finger nucleases (ZFNs), transcription activator-like effector nucleases (TALENs) and the clustered regularly interspaced short palindromic repeats (CRISPR)/CRISPR associated protein 9 (Cas9) system, have allowed researchers to generate diverse genomic modifications in whole animals and in cultured cells. The CRISPR/Cas9 and TALEN techniques frequently induce DNA double-strand breaks (DSBs) at the targeted gene, resulting in frameshift-mediated gene disruption. As a useful application of genome editing technology, several groups have recently reported efficient site-specific integration of exogenous genes into targeted genomic loci. In this review, we provide an overview of TALEN- and CRISPR/Cas9-mediated site-specific integration of exogenous genes in zebrafish.

## 1. Introduction

The zebrafish is a useful model organism for basic biology and applied research, including *in vivo* drug screening, owing to its remarkable characteristics, such as its small size, rapid generation time and optical transparency during early embryogenesis [1,2]. Both knockdown and knockout analyses are useful for examining the *in vivo* function of uncharacterized genes. The injection of antisense morpholino oligonucleotides (MO) in zebrafish embryos, which causes suppression of translation of the target gene, is generally used to knock down the endogenous target gene [3]. However, undesirable off-target effects of MO injection have been reported and MO injection in zebrafish embryos frequently gives rise to the ectopic induction of p53 [4]. In the case of knockout analysis, genome editing technologies efficiently induce DNA double-strand breaks (DSBs) in the targeted gene, leading to frameshift-mediated gene disruption [5,6]. Two groups firstly reported the zinc finger nuclease (ZFN)-mediated gene disruptions in zebrafish [7,8]. Recent accumulating evidence shows that genetically gene-disrupted mutants and morphants (MO-injected embryos) often exhibit distinct phenotypes [4,9,10,11]. The complete suppression of maternal factors by MO is difficult, limiting the use of MO-based knockdown analysis. The functional analysis of zebrafish maternal-zygotic mutants established through genome editing technologies revealed novel developmental functions of maternal factors [12,13]. Therefore, these technologies are indispensable for the loss-of-function analysis of maternal and/or zygotic factors in zebrafish.

Genome editing technologies (ZFN, transcription activator-like effector nuclease (TALEN) and clustered regularly interspaced short palindromic repeats (CRISPR)/CRISPR associated protein 9 (Cas9) enable us to manipulate various genomic modifications in model organisms and in cultured cells [5,14,15,16]. Both ZFNs and TALENs are chimeric proteins fusing the DNA-binding domains required for the protein-DNA interaction and the *Fok*I nuclease catalytic domain [17,18,19]. Because *Fok*I nuclease functions as a dimer, DSBs are produced in the spacer region located between the DNA recognition sites of a pair of ZFNs or TALENs. A ZFN possesses four to six Cys_2_-His_2_ zinc finger motifs, one of which recognizes three nucleotides, and each zinc finger domain often interferes with nucleotide recognition specificity [20]. Each TALEN possesses 14.5–19.5 TALE effector repeats, one of which independently recognizes a single nucleotide. Each TALE repeat comprises 34 highly conserved residues except the two repeat-variable di-residues (RVDs) at amino acid positions 12 and 13. Therefore, the RVDs are essential for the nucleotide recognition specificity known as the TALE code: NG = T, HD = C, NI = A, NN = G or A. Efficient assembly methods for ordering TALE repeats have been developed [18,19,21,22]; therefore, the construction of functional TALENs is much easier than the construction of functional ZFNs. Recently, the CRISPR/Cas9 system has emerged as a more convenient technique for genome editing [15,23,24]. The type 2 CRISPR/Cas9 system originally consisted of three components: a Cas9 nuclease and two short RNAs, the CRISPR RNA (crRNA) and the *trans*-activating crRNA (tracrRNA) [15]. The crRNA is essential for the RNA–DNA interaction and possesses a complementary stretch of RNA (20 bases) to generate specificity to the targeted genome sequences followed by the protospacer-adjacent motif (PAM) sequence NGG (N: any nucleotide). The tracrRNA is required to interact with the crRNA and Cas9 nuclease. We and another group have reported a ready-to-use method consisting of synthetic crRNA, synthetic tracrRNA and recombinant Cas9; this complex cleaves the targeted genomic locus with high frequency [25,26]. Importantly, a single guide RNA (gRNA), combining the crRNA and tracrRNA, was elegantly developed and has been widely used as a simplified two-component system [23,24]. More recently, Zetsche *et al.* reported a novel class 2 CRISPR effector, Cpf1, which is a single RNA-guided endonuclease [27].

**Figure 1:**
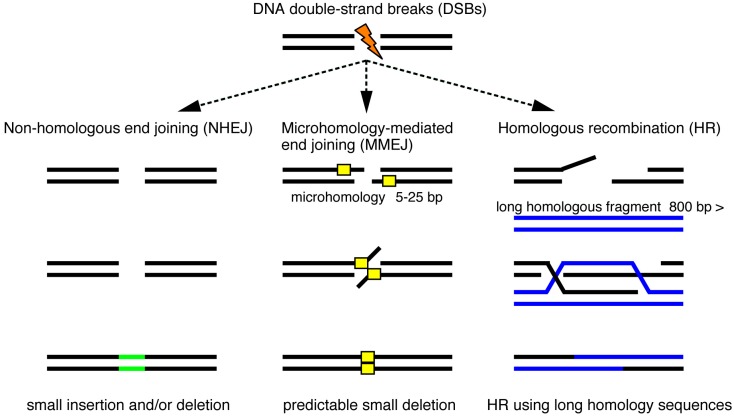
Targeted genomic modifications using genome editing technologies. DNA double-strand breaks (DSBs) induced by genome editing technologies are repaired by non-homologous end joining (NHEJ), microhomology-mediated end joining (MMEJ) and homologous recombination (HR). NHEJ repair, which connects the ends of the broken strands, leads to unpredictable insertion and/or deletion mutations (green bar), while MMEJ repair uses microhomology sequences (yellow box) and often causes a predictable small deletion. HR repair requires long double-strand DNA fragments (blue bar) that possess homology to the targeted genomic locus. Site-specific integrations of donor DNA are mediated by these DNA repair mechanisms.

**Figure 2:**
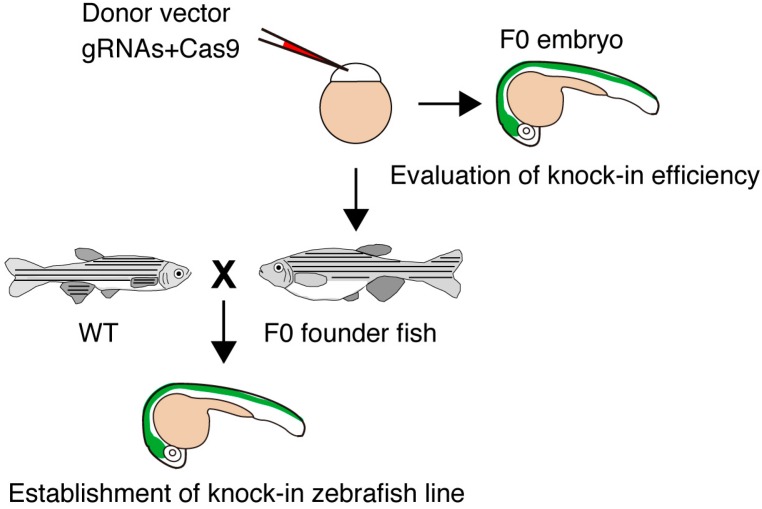
Strategy for establishing knock-in fish. Donor vector, gRNAs and Cas9 mRNA are injected into zebrafish embryos. The knock-in event is estimated by examining the expression of the fluorescent gene (green area). Potential F0 founders are mated with wild-type (WT) fish, and the knock-in lines expressing the fluorescent gene are selected. The targeted knock-in at the targeted locus is determined by genomic PCR and sequencing analysis.

Genome editing technologies efficiently produce site-specific DSBs that are usually repaired by non-homologous end joining (NHEJ), microhomology-mediated end joining (MMEJ) and homologous recombination (HR) (Figure 1) [28,29]. In the absence of donor DNA templates, the error-prone NHEJ pathway directly connects the ends of broken strands, leading to the production of insertion and/or deletion (indel) mutations. DSBs can be repaired by HR in the presence of donor DNA templates with large homology to the target site. Recently, an alternative end-joining pathway was characterized; MMEJ joins the exposed ends at microhomology regions (five to 25 bases) on the target locus, which are annealed and filled in by DNA polymerases. In mouse or rat, the HR-mediated knock-in of homologous fragments derived from a donor vector functions well. However, HR-dependent knock-in events are restricted in zebrafish as described below. One possible explanation of such a difference is that initial mitoses of zebrafish embryonic cleavages occur faster than those of mouse. Here, we review two HR-independent knock-in technologies, NHEJ- and MMEJ-mediated targeted integrations of exogenous genes, in zebrafish (Figure 2).

## 2. Genomic Insertion of Single-Stranded Oligodeoxynucleotides (ssODNs)

It has been shown that short oligonucleotide templates, called single-stranded oligodeoxynucleotides (ssODNs), can be used to introduce genomic alterations, including single nucleotide substitutions, to model organisms and cultured cells [30,31]. Using TALENs and the CRISPR/Cas9 system, ssODNs can be integrated at a targeted genomic locus in zebrafish [30,32,33,34]. Donor ssODNs possess approximately 20 to 50 base homologous sequences to the target site and the designed DNA fragments, such as loxP, restriction enzyme sites and hemagglutinin (HA) tags. Precise genomic alteration with ssODNs can be introduced at the targeted loci, whereas undesired mutations, including indel mutations and tandem integration of ssODNs, are simultaneously observed [30,32,33]. Interestingly, Yoshimi *et al.* demonstrated that the CAG-GFP donor vector was efficiently integrated into the rat *Rosa26* locus using two ssODNs (80 bases) that bridged the linearized donor vector and the targeted genomic locus [31]. It is noteworthy that site-directed genomic modifications and donor DNA insertion using ssODNs are useful to establish model organisms for human genetic diseases, which includes the various mutations responsible for genetic disorders.

## 3. Precise Site-Specific Integration of Donor DNA by Homologous Recombination (HR)

Genome editing technologies (TALEN and CRISPR/Cas9) were applied to perform HR-mediated integration of donor DNA templates because the HR of the donor vector at the target site is largely enhanced by the production of site-specific DSBs. The eGFP reporter donor containing approximately 1 kb of homology arms was integrated at the target site using HR in zebrafish [35]; however, its efficacy for knock-in use was relatively low to put into practical use. In this report, the authors confirmed the germline transmission of the knock-in allele at the *tyrosine hydroxylase* locus in four of 275 potential F0 zebrafish founders. Subsequently, Shin *et al.* reported the efficient TALEN-mediated knock-in of donor DNA templates by optimizing the length of the homology arms and the configuration of the donor DNA construct [36]. They used various sizes of homology arms (ranging from 0.3 to 3.7 kb) in the donor vectors and found that the long homology arms (more than 2 kb) may be sufficient to achieve the HR-mediated knock-in. Additionally, superfolder GFP (sfGFP) was integrated by TALENs into the *sox2* locus, and 29 of 363 F0 founders produced sfGFP-positive F1 embryos. Furthermore, tandem dimeric Tomato (tdTomato) was integrated into the *gfap* locus, and five of 44 F0 founders produced tdTomato-positive F1 embryos [36]. More recently, Irion *et al.* reported the CRISPR/Cas9-mediated knock-in of a donor vector [37]. To examine the knock-in efficacy, they chose the *albino* mutant (*alb^b4^*), which carries a nonsense mutation in the *slc45a2* gene essential for melanin production. Because the *alb* mutant embryos exhibit a pale phenotype with pigment defects, the phenotypic rescue by the HR-mediated knock-in of a donor vector containing the wild-type *slc45a2* gene is easily determined by the appearance of the pigmented cells. They demonstrated that the frequency of phenotypic rescue was increased using circular donor DNA containing CRISPR target sites. In the rescue experiment, three of 28 F0 founders produced pigmented embryos with the wild-type phenotype [37]. Although the construction of donor vectors containing long homology arms is very complicated and time-consuming, HR-mediated genome editing in zebrafish is a powerful tool for precise genomic modifications.

## 4. Genomic Insertion of Donor DNA by Non-Homologous End Joining (NHEJ)

Recently, site-specific insertion via homology-independent repair mechanisms, presumably mediated by NHEJ, was developed. When the targeted genomic locus and the donor vector containing the ZFN or TALEN target sequences were simultaneously cleaved by ZFN or TALEN, the NHEJ-mediated insertion of donor DNA was observed in cultured cells at the target site [38,39]. One advantage of this method is the simple construction of the donor vector because there is no requirement for long homology arms. Auer *et al.* reported the efficient CRISPR/Cas9-mediated knock-in of donor vectors at targeted sites in zebrafish [40]. In this case, the donor vector contained a bait sequence efficiently cleaved by CRISPR/Cas9. When the donor vector containing a CRISPR target site was co-injected with gRNAs and Cas9 mRNA, concurrent cleavage of the donor vector and the targeted genomic locus caused the efficient genomic insertion of the donor vector at the target site. The authors targeted the eGFP locus in an eGFP transgenic line, and the donor vector containing the bait sequence and the transcriptional activator Gal4 gene was co-injected with gRNAs and Cas9 mRNA into the embryos containing the eGFP and UAS promoter-RFP transgenes. Successful in-frame Gal4 integration, but not out-of-frame and reverse integration, was easily visualized by a switch from eGFP to RFP expression. The authors demonstrated that the germline transmission rate of forward donor DNA integration was 31% (9 of 29 F0 founders) [40]. When they selected the embryos expressing RFP and allowed them to grow into adult fish, the germline transmission rate of in-frame integration increased to 40% (2 of 5 F0 founders). It should be noted that the ratio of in-frame integration of the donor vector is theoretically one-sixth of all integration events because out-of-frame and reverse integrations are not functional.

**Figure 3:**
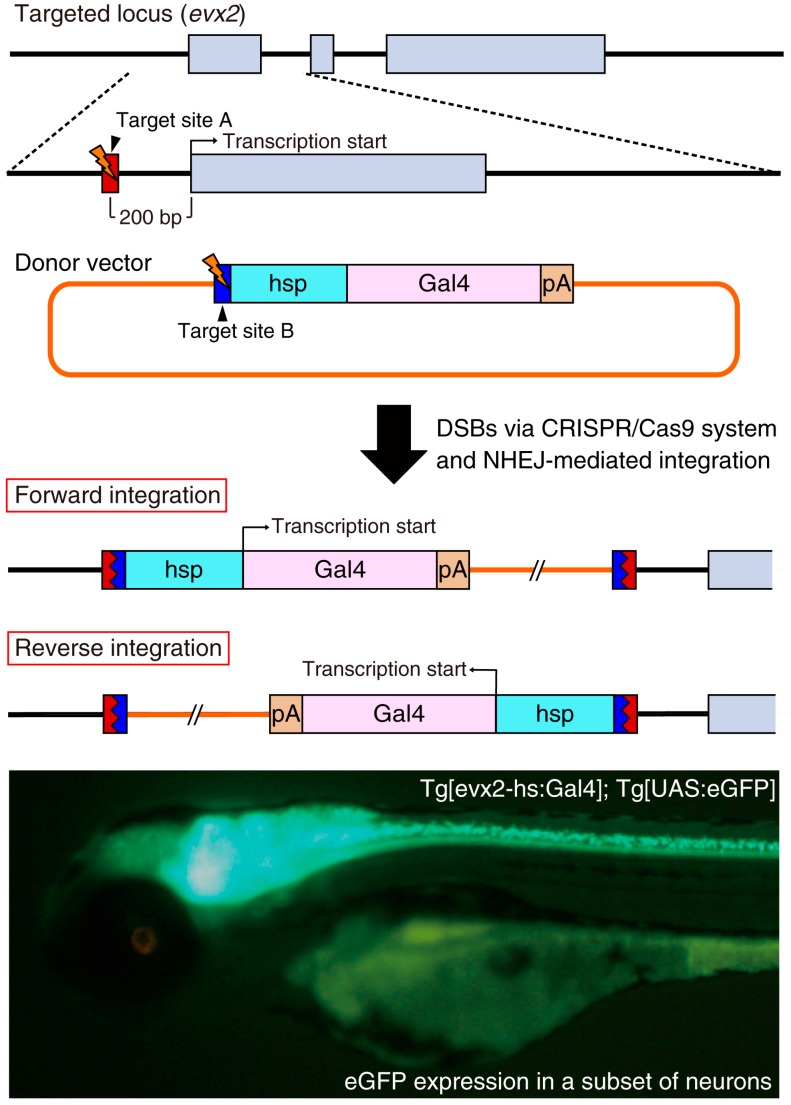
Site-specific insertion of the hsp promoter-Gal4 donor into the *evx2* locus. The donor vector consists of a bait sequence (blue box; target site B for Gbait-gRNA), the hsp promoter (light blue box), a Gal4 driver (pink box) and a polyA signal (pA) (orange box). The target site A for evx2-gRNA (red box) is located in the promoter region of the *evx2* gene. When the CRISPR/Cas9 system simultaneously cleaves the target site A and B, the Gal4 driver is unpredictably integrated into the *evx2* locus. Not only forward and reverse integrations but also tandem donor vector integrations can occur. We observed eGFP expression in a subset of neurons of the Tg[evx2-hs:Gal4]; Tg[UAS:eGFP] transgenic line because Gal4 driven by the endogenous *evx2* enhancer induced eGFP expression as described previously [41]. Gray boxes; exons in the *evx2* gene.

More recently, Kimura *et al.* improved the NHEJ-mediated knock-in of donor vectors for establishing transgenic zebrafish [41]. They prepared the donor vector containing a heat shock protein 70 (hsp) promoter and reporter/driver genes (fluorescent protein genes or the Gal4 gene) (Figure 3) and designed gRNA targeting 200–600 bp upstream of the target gene transcription start site. Because the hsp promoter in the donor DNA can receive the enhancer activity of the endogenous target gene, both forward and reverse genomic integration of the donor DNA should be functional. In fact, they established the Tg[evx2-hs:Gal4]; Tg[UAS:eGFP] knock-in line in the reverse direction and the eGFP expression was comparable to the endogenous expression of the *evx2* gene. Furthermore, the introduction of the hsp promoter usually enhances the basal expression of the integrated reporter/driver gene. Immunostaining using an anti-Evx2 antibody revealed that the eGFP expression generally overlapped with Evx2 protein expression. However, the expression of the Evx2 protein was not detected in some of the eGFP-positive cells, presumably owing to the enhanced leaky expression of the hsp-dependent promoter activity. Using this strategy, the authors consistently established stable knock-in lines in four endogenous loci (*evx2*, *eng1b*, *glyt2* and *vglut2a*) [41]. Importantly, the donor vector containing the reporter/driver gene is acceptable for genomic integration of any target genes. This makes this technique very convenient compared with the conventional BAC (bacterial artificial chromosome) method that requires complicated and tailored BAC constructs to individual target genes [42]. The CRISPR/Cas9-mediated knock-in of donor DNA becomes a powerful genetic tool for the generation of transgenic animals with cell-type-specific gene expression.

## 5. Precise Targeted Integration of Donor DNA by Microhomology-Mediated End Joining (MMEJ)

**Figure 4:**
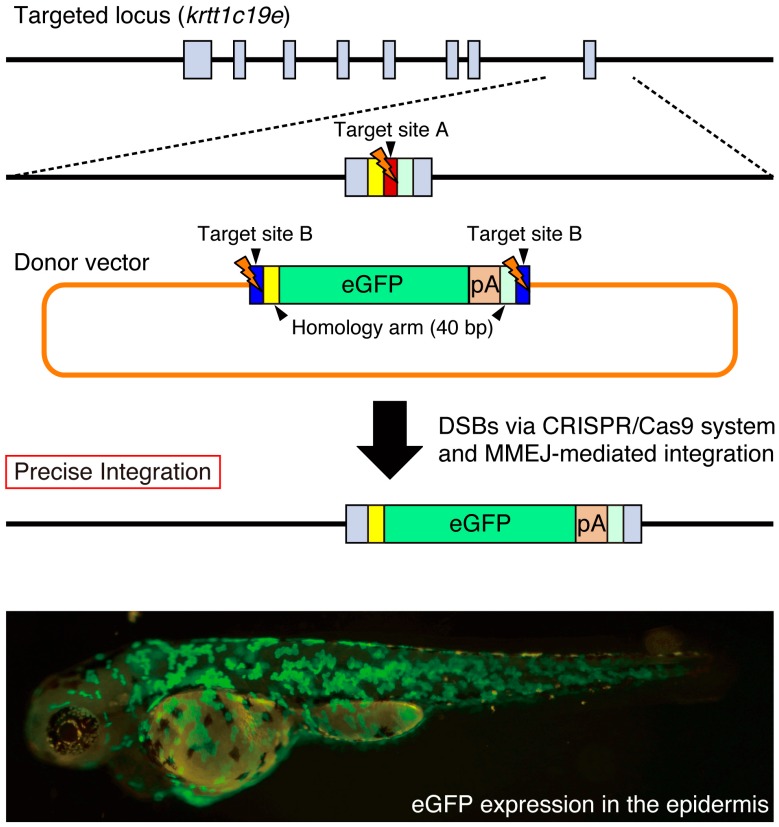
Precise site-specific integration of eGFP into the *krtt1c19e* locus. The donor vector consists of two bait sequences (blue box; target site B for Gbait-gRNA), a left homology arm (yellow box; 40 bp), and an eGFP (green box) and polyA signal (pA) (orange box) and right homology arm (light green box). The target site A for krtt1c19e-gRNA (red box) is located near the stop codon of the *krtt1c19e* gene. When the CRISPR/Cas9 system simultaneously cleaves the target sites A and B, the eGFP reporter between the microhomology arms is precisely integrated into the *krtt1c19e* locus. Broad eGFP expression was detected in the epidermis of the injected F0 embryo as described previously [44]. Gray boxes; exons in the *krtt1c19e* gene.

It has been recently shown that DSBs can be repaired by MMEJ that uses the existing microhomology sequences (five to 25 bases) around the broken DNA ends [29]. Both Dr. Yamamoto’s group and our group have developed novel knock-in methods acceptable for the precise targeted integration of exogenous genes mediated by the MMEJ pathway [43,44,45]. Using TALEN or the CRISPR/Cas9 system, they have succeeded in the MMEJ-mediated knock-in of donor vectors in cultured cells and in animals, including silkworms and frogs [43,45]. We designed a more versatile donor vector, which possesses a bait sequence (a functional gRNA target sequence) and short homology arms (10–40 bp) flanking the targeted genomic locus. We prepared a control vector without homology arms to examine the NHEJ-mediated knock-in of the donor vector. In the case of the *tyrosinase* locus, the NHEJ-mediated knock-in of the donor vector in the forward direction was detected in 53% of the injected F0 embryos using PCR, while the addition of 10–40 bp homology arms in the donor vector increased its efficiency to 85%. Genomic sequence analysis revealed that the MMEJ-mediated knock-in with precise targeted integration was 60%–77% of analyzed knock-in events. Therefore, the introduction of short homology arms promotes precise targeted integration of exogenous genes. In order to eliminate the undesired integration of the backbone vector sequences, we designed an improved donor vector that configures the eGFP and polyA signal between two bait sequences in the case of the *keratin type 1 c19e/krtt1c19e* locus (Figure 4). We observed eGFP expression in the epidermis at a high frequency (201/529 injected F0 embryos). We established stable zebrafish lines expressing eGFP in the epidermis, indicating that the MMEJ-mediated targeted integration of the exogenous gene is heritable. We found that the pre-screening of F0 embryos exhibiting appropriate eGFP expression is beneficial for the efficient identification of the knock-in allele. We propose that the MMEJ-mediated targeted integration of exogenous genes is a powerful genetic tool for precise genomic modifications in various model organisms.

## 6. Concluding Remarks and Future Perspective

Genetic manipulation in whole organisms plays an important role in basic, applied and medical sciences. Recent advances in genome editing technologies (ZFN, TALEN and CRISPR/Cas9) have enabled researchers to make precise genomic modifications that were previously difficult or impossible in various organisms including humans. In this review, we summarized the targeted integration of exogenous genes using genome editing technologies mediated by DNA repair mechanisms, such as NHEJ, MMEJ and HR. Because integration directions become random and various indel mutations occur at the junction in the process of NHEJ-mediated knock-in, we recommend that MMEJ-mediated knock-in technology is suitable for the precise targeted integration of donor DNA templates. Furthermore, the construction of a donor vector for MMEJ-mediated knock-in is much easier than that of HR-mediated knock-in. Therefore, we predict that the MMEJ-mediated targeted integration could be widely applied in various organisms. Importantly, genome editing technologies can be used to precisely model human genetic disorders in animals including zebrafish, which could lead to novel findings with respect to the molecular mechanisms of human diseases. Furthermore, these technologies make it easier to correct serious genetic disorders in affected patients and in induced pluripotent stem (iPS) cells derived from patients. Further innovation of genome editing technologies will accelerate various applications in basic biology, biotechnology and medicine, including potential therapeutic strategies for human genetic disorders.

## Abbreviations

ZFN	zinc finger nuclease
TALEN	transcription activator-like effector nuclease
CRISPR	clustered regularly interspaced short palindromic repeats
MO	morpholino oligonucleotide
RVD	repeat-variable di-residue
crRNA	CRISPR RNA
tracrRNA	*trans*-activating crRNA
PAM	protospacer-adjacent motif
gRNA	guide RNA
DSB	double-strand break
NHEJ	non-homologous end joining
MMEJ	microhomology-mediated end joining
HR	homologous recombination
indel	insertion and/or deletion
ssODN	single-stranded oligodeoxynucleotide
HA	hemagglutinin
BAC	bacterial artificial chromosome
iPS	induced pluripotent stem
